# Sleep-Related Breathing Disorders and Pregnancy: Where We Stand and Where to Go

**DOI:** 10.3390/medicina62050835

**Published:** 2026-04-28

**Authors:** Jorge Montês, Mónica Grafino, Miguel Ângelo-Dias, Jorge Lima, Sofia Tello Furtado

**Affiliations:** 1 Pulmonology Department, Hospital da Luz Lisboa, 1500-650 Lisbon, Portugal; jorge.ambrosio.montes@hospitaldaluz.pt (J.M.); monica.grafino@hospitaldaluz.pt (M.G.); sfurtado@hospitaldaluz.pt (S.T.F.); 2 Comprehensive Health Research Centre—CHRC, NOVA Medical School, Faculdade de Ciências Médicas, NMS, FCM, Universidade NOVA de Lisboa, 1169-056 Lisbon, Portugal; miguel.dias@nms.unl.pt; 3 Gynecology and Obstetrics Department, Hospital da Luz Lisboa, 1500-650 Lisbon, Portugal

**Keywords:** pregnancy, obstructive sleep apnea, sleep-related breathing disorders

## Abstract

*Background and Objectives*: Pregnancy causes various physiological and hormonal changes that disrupt sleep architecture and modify respiratory patterns, increasing the risk of sleep-related breathing disorders (SBDs) such as obstructive sleep apnea (OSA) and potentially exacerbating pre-existing conditions. These disorders have been linked to adverse maternal and fetal outcomes. However, current screening tools remain inadequate, and data, including from Portugal, remain limited. This study aimed to assess the prevalence of SBD symptoms suggestive of sleep-disordered breathing during pregnancy, characterize the population, and explore associations with demographic and anthropometric parameters. *Materials and Methods*: A prospective observational study was conducted from July to December 2024 at Hospital da Luz Lisboa, involving pregnant women ≥ 18 years attending routine consultations. Participants completed a structured questionnaire that assessed demographic and anthropometric data, comorbidities, ten SBD symptoms, and the Epworth Sleepiness Scale (ESS). *Results*: The cohort included 289 participants, with a mean age of 34.4 years and pre-pregnancy body mass index (BMI) of 23.6 kg/m^2^. On average, women reported 3.1 SBD symptoms, with fatigue (65.4%), memory/concentration impairment (52.2%), and non-restorative sleep (50.5%) being the most common. Excessive daytime sleepiness (ESS >10) was present in 22.8% of the population. Snoring was significantly associated with older age and higher BMI (*p* = 0.0009 and *p* < 0.0001, respectively). Both the number of symptoms and ESS scores tended to increase with gestational age, particularly in the third trimester. Women with diabetes had higher odds of reporting snoring, nocturnal dyspnea, and witnessed apneas, with odds ratios of 4.65, 8.77, and 11.38, respectively. *Conclusions*: SBD symptoms and daytime sleepiness are highly prevalent in pregnancy and typically increase with advancing gestation. These findings emphasize the need for improved clinical strategies to enable early identification and management of SBD in pregnant women, thereby reducing maternal-fetal complications.

## 1. Introduction

Pregnancy entails substantial physiological and hormonal changes that can modify sleep architecture and respiratory function. These alterations increase the risk of developing sleep-related breathing disorders (SBDs) or exacerbating pre-existing respiratory conditions [1,2].

SDB encompasses a range of conditions from simple snoring to obstructive sleep apnea (OSA), the latter of which can manifest with nonspecific symptoms during pregnancy. The exact prevalence of SBDs among pregnant populations is uncertain and varies widely. This variability depends on several factors, including study population characteristics, sample size, pregnancy stage, risk profile, and the type of diagnostic tools employed [3,4].

Studies show that snoring is prevalent during pregnancy, affecting up to 35% of pregnant women [5]. Additionally, the prevalence of OSA tends to rise as pregnancy advances, with estimates ranging from 10.5% in the first trimester to 26.7% in the third trimester. Significant risk factors for OSA in pregnancy include a high pre-pregnancy body mass index (BMI) and advanced maternal age [6].

These disorders are linked to various adverse maternal and fetal outcomes. Recently published evidence from systematic reviews and meta-analyses has further reinforced the clinical relevance of sleep-disordered breathing during pregnancy, highlighting its association with adverse maternal and fetal outcomes, including gestational diabetes, hypertensive disorders, preterm birth, and fetal growth restrictions [3,4,7,8,9,10]. Despite these risks, screening for SBDs during pregnancy remains challenging. Commonly used questionnaires, such as Berlin, STOP-BANG, and the Epworth Sleepiness Scale (ESS), lack validation and have demonstrated low sensitivity and specificity in this population [8,9,10,11,12,13,14].

Polysomnography is the gold standard for diagnosing SBD; however, its clinical use, especially among pregnant women, faces limitations due to practical constraints such as restricted access, high costs, and the need for timely results [11,12,13].

Furthermore, there is a lack of scientific literature in Portugal that addresses and evaluates this reality in the pregnant population.

This study aimed to determine the prevalence of self-reported SBD symptoms during pregnancy and explore potential associations between these symptoms and various demographic or anthropometric factors.

## 2. Methods

### 2.1. Subjects and Protocol

In this prospective observational study, pregnant women were recruited during routine obstetric appointments between July and December 2024. The study took place at Hospital da Luz Lisboa, a large private tertiary hospital in Lisbon, Portugal, which manages nearly 4000 deliveries each year. Inclusion criteria required participants to be at least 18 years old and to have completed a questionnaire and signed a consent form for the study. Exclusion criteria encompassed women who refused or were unable to consent and those with a previously diagnosed SBD [3].

All participants attended routine antenatal visits at Hospital da Luz Lisboa, receiving standard care based on their obstetrician’s clinical judgment.

The Ethics Committee of Hospital da Luz Lisboa approved the study protocol (ID 609, CES 03/2024), and written informed consent was obtained from all participants.

The questionnaire gathered demographic and clinical data, specifically age (in years), gestational age (in weeks), height, and weight (both pre-pregnancy and at the time of questionnaire completion), as well as pre-pregnancy BMI (kg/m^2^). It also evaluated medical history and comorbidities. Additionally, the questionnaire assessed self-reported SBD-related symptoms, including fatigue, memory and concentration impairment, non-restorative sleep, insomnia, daytime sleepiness, snoring, nocturnal movements, behavioral changes, nocturnal dyspnea, and witnessed apneas, as well as the ESS [3].

Nocturnal dyspnea was defined as self-reported episodes of shortness of breath occurring during the night. Behavioral changes were defined as self-reported alterations in mood, irritability, or abnormal nocturnal behaviors noticed by the patient or reported by a partner.

The ESS is a widely used instrument to assess daytime sleepiness. It comprises eight questions that evaluate an individual’s likelihood of falling asleep in various situations. The ESS scores range from 0 to 24 points, with values greater than 10 indicating excessive daytime sleepiness [12].

### 2.2. Statistical Analysis

Categorical variables are reported as absolute frequencies and percentages. Associations between these variables were analyzed using the chi-square or Fisher’s exact test, as appropriate. We assessed data normality through visual inspection using histograms and QQ plots of the residuals, utilizing the D’Agostino–Pearson normality test when necessary. Normally distributed data are presented as mean and standard deviation (SD), while non-normally distributed data are presented as median and range. To compare two independent groups, we applied either the Unpaired *t*-test or the Mann–Whitney test, as suitable. For determining statistical significance among more than two independent groups, we used the Kruskal–Wallis test followed by Dunn’s multiple comparisons test.

To explore cross-sectional differences in the frequency of sleep-related symptoms and global sleep disruption scores throughout pregnancy, we employed centered linear or polynomial regression curves.

For all analyses, a two-tailed *p*-value of <0.05 was deemed significant. Statistical analyses and visualizations were conducted using GraphPad Prism v10.4.0 for Windows (GraphPad Software, Boston, MA, USA, https://www.graphpad.com).

## 3. Results

The cohort consisted of 289 pregnant individuals, with their demographic characteristics outlined in Table 1. The mean maternal age was 34.4 years (SD = 4.9), with 16.3% of participants aged 40 years or older. The mean gestational age at the time of questionnaire completion was 21.5 weeks (SD = 9.1), ranging from a minimum of 5 weeks to a maximum of 37 weeks. The mean pre-pregnancy BMI was 23.6 kg/m^2^ (SD = 4.1).

The majority of the pregnant women were nulliparous (43.6%) and in their second trimester (45.0%). Clinically relevant comorbidities included respiratory diseases (31 cases—10.7%), primarily asthma (n = 23, 7.9%). Hypertension was present in 14 cases (4.8%), and diabetes in 10 cases (3.5%).

Participants reported an average of 3.1 symptoms of SBD, with a median of 3, and a range of 0 to 7. Excessive daytime sleepiness was observed in 65 women, accounting for 22.8% of participants.

### 3.1. SBD Symptoms and Their Associations

Table 2 reveals that the most commonly reported symptoms related to SBD were fatigue (65.4%), memory and concentration impairment (52.2%), and non-restorative sleep (50.5%).

A comparative analysis of participants with and without specific symptoms, considering ESS scores, maternal age, gestational age, and pre-pregnancy BMI (Table S1, Supplementary Material), revealed several statistically significant associations. Fatigue, memory/concentration impairment, non-restorative sleep, nocturnal dyspnea, and reported daytime sleepiness were linked to higher ESS scores (all *p* < 0.0001). Participants experiencing non-restorative sleep and memory/concentration impairment also exhibited higher maternal ages (*p* = 0.0185 and *p* = 0.0199, respectively). Snoring was associated with both advanced maternal age (mean = 36.0 vs. 33.8 years; *p* = 0.0009) and a higher pre-pregnancy BMI (mean = 25.7 vs. 22.9 kg/m^2^; *p* < 0.0001). Nocturnal dyspnea correlated with significantly increased ESS scores (*p* = 0.048) and showed a trend toward higher gestational age. No statistically significant associations were identified for the remaining symptoms.

### 3.2. Cross-Sectional Distribution of Sleep-Related Symptoms and ESS Across Gestational Age

**Figure 1:**
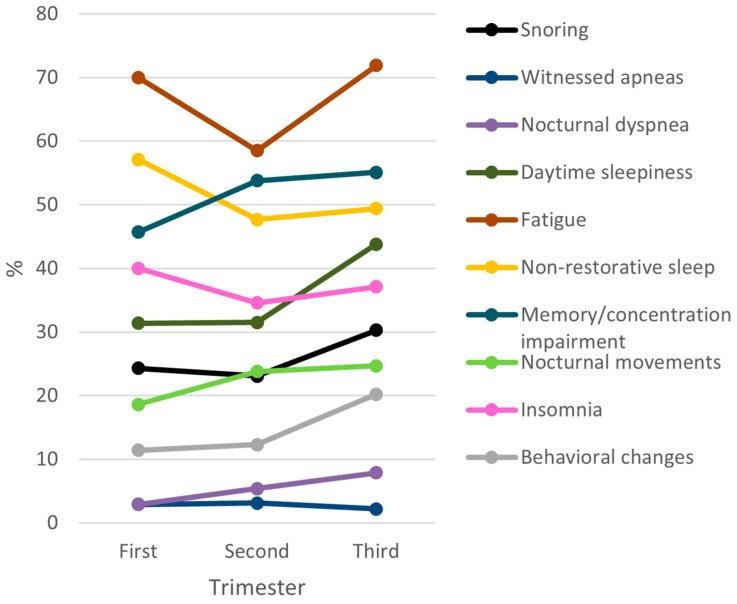
Cross-sectional distribution of sleep-related symptoms across gestational age.

The frequency of each SBD symptom was analyzed across pregnancy trimesters (Figure 1). A visual inspection of symptom distribution across trimesters suggests a higher frequency of several symptoms in the third trimester, particularly fatigue, non-restorative sleep, and daytime sleepiness.

**Figure 2:**
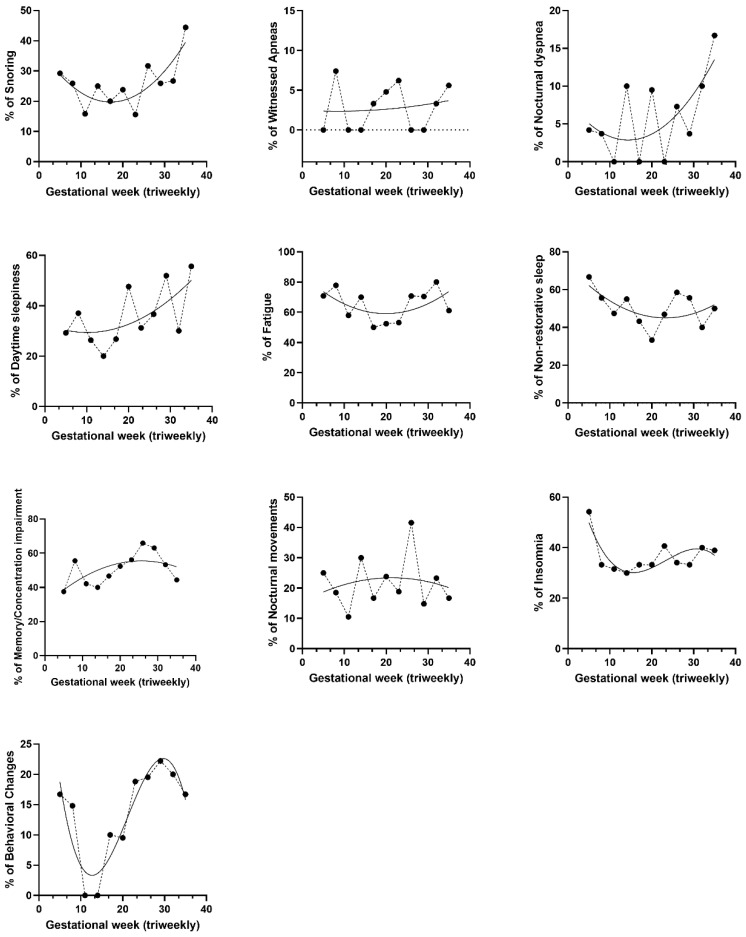
Cross-sectional frequency of reported sleep-related symptoms across gestational weeks. Each plot displays the percentage of participants reporting a given symptom, grouped in 3-week gestational intervals. Curves represent fitted centered second-order polynomial trend lines for each symptom, except for insomnia and behavioral changes, which indicate centered third-order polynomial curves.

To explore the cross-sectional distribution of sleep-related symptoms according to gestational week, researchers plotted the frequency of each symptom for each gestational week, grouped into 3-week intervals (Figure 2). The analysis revealed a variety of patterns. Fatigue, non-restorative sleep, and reported daytime sleepiness exhibited a U-shaped distribution, with increased prevalence in both early and late gestation. Conversely, memory and concentration impairments, along with insomnia, showed a consistent upward distribution throughout pregnancy. Snoring and nocturnal dyspnea appeared more frequent among participants assessed in later gestational stages, particularly in the third trimester. Other symptoms, such as nocturnal movements and behavioral changes, fluctuated throughout pregnancy without exhibiting a consistent pattern.

**Figure 3:**
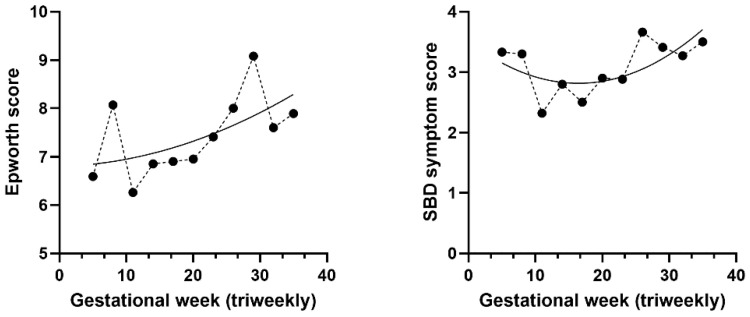
Cross-sectional distribution of mean ESS and symptom score across gestational week groups. Mean scores for the ESS (**left**) and SBD symptom score (**right**) are plotted by gestational week, grouped in 3-week intervals. Each dot represents the group mean, and the line represents the fitted centered second-order polynomial trend.

In addition to examining the frequency of individual symptoms, we analyzed the cross-sectional distribution of global sleep disruption scores throughout pregnancy. Both the ESS score and the SBD symptom score, which represent the total number of reported sleep-related symptoms, exhibited higher values in the group of pregnant women with later gestational stages in pregnancy (Figure 3).

The data, when grouped in 3-week intervals, indicated a modest increase in ESS scores over time. Similarly, the SBD symptom score was higher in later gestational week groups. However, analysis by trimester revealed no significant differences in either score.

**Figure 4:**
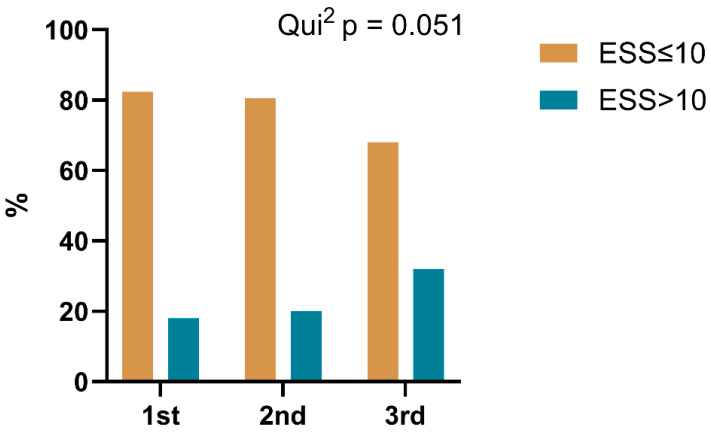
Changes in Epworth Sleepiness Scale (ESS) by trimester. Bars indicate the proportion of participants with an ESS score greater or lower than 10 in each trimester.

Examining excessive daytime sleepiness as measured by the ESS, the proportion of participants with an ESS score greater than 10 increased from 18% in the first trimester to 32% in the third trimester. This change suggests a trend toward statistical significance (*p* = 0.051) (Figure 4).

### 3.3. Symptom Frequency and Sleep Scores by Maternal Age Group

We conducted an additional analysis of the frequency of SBD-related symptoms and scores, categorizing maternal age into four groups (Table 1). Although no symptoms achieved statistical significance, snoring (*p* = 0.059) and non-restorative sleep (*p* = 0.066) approached significance, indicating potential trends (Table S2, Supplementary Material).

**Figure 5:**
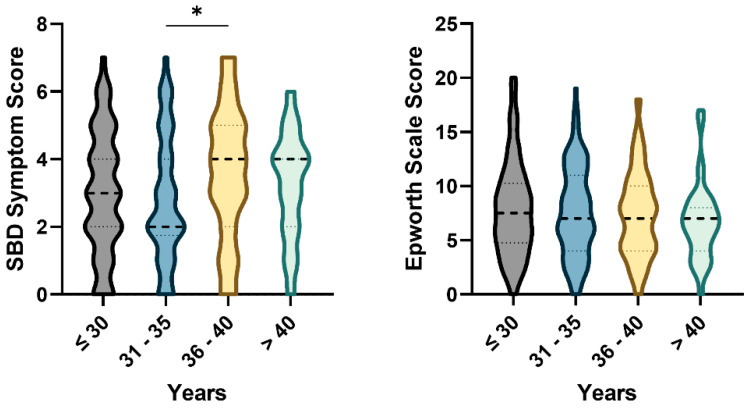
SBD symptoms and ESS scores across maternal age groups. Violin plots display the distribution of scores across age groups (≤30, 31–35, 36–40, >40 years). Horizontal dashed lines represent median values with interquartile range. Statistical significance between groups was determined with the Kruskal–Wallis test, followed by Dunn’s multiple comparisons test, and is indicated as * *p* < 0.05. SBD, Sleep breathing disorder; ESS, Epworth Sleepiness Scale.

The SBD symptom score varied significantly among different maternal age groups (*p* < 0.05), with women aged 36–40 years exhibiting the highest median score compared to younger groups. In contrast, ESS scores showed no significant differences across age groups (Figure 5).

### 3.4. Symptom Frequency and Sleep Scores by Pre-Pregnancy BMI

We further analyzed the frequency of SBD-related symptoms and scores across four BMI categories: underweight (<18.5 kg/m^2^), normal weight (18.5–24.9 kg/m^2^), overweight (25.0–29.9 kg/m^2^), and obese (≥30 kg/m^2^).

**Figure 6:**
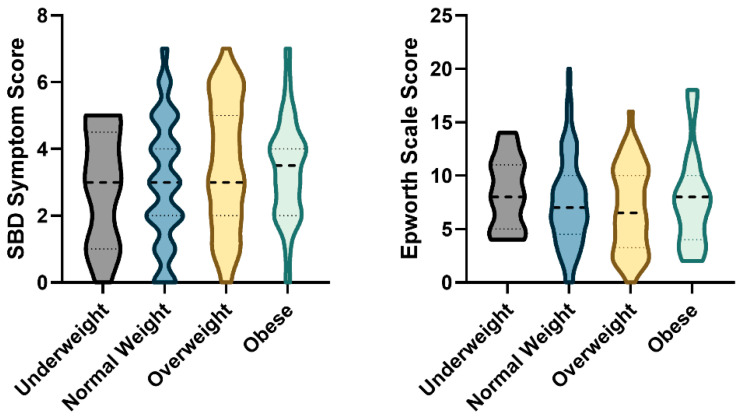
SBD symptoms and ESS scores across BMI groups. Violin plots display the distribution of scores across BMI groups [underweight (<18.5 kg/m^2^), normal weight (18.5–24.9 kg/m^2^), overweight (25.0–29.9 kg/m^2^), and obese (≥30 kg/m^2^)]. Horizontal dashed lines represent median values with interquartile range. Statistical significance between groups was determined with the Kruskal–Wallis test followed by Dunn’s multiple comparisons test. SBD, Sleep breathing disorder; ESS, Epworth Sleepiness Scale.

When comparing the distribution of sleep symptoms across BMI categories, snoring exhibited a significant association (*p* < 0.001). Its prevalence increased progressively from 7.7% in the underweight group to 53.8% in the obese group. In contrast, no statistically significant differences were observed for the other sleep symptoms (Table S3, Supplementary Material), nor for the SBD symptom score or ESS score (Figure 6) across BMI groups.

### 3.5. Symptom Associations with Comorbidities

No significant differences in ESS or SBD symptom scores were associated with the presence of comorbidities overall. However, among participants with diabetes, the likelihood of specific SBD-related symptoms was significantly higher. Compared to non-diabetic women, those with diabetes had substantially higher odds of reporting snoring (OR = 4.65; 95% CI: 1.28–16.98; *p* = 0.020), witnessed apneas (OR = 11.38; 95% CI: 1.98–65.32; *p* = 0.027), and nocturnal dyspnea (OR = 8.77; 95% CI: 2.03–37.86; *p* = 0.013).

## 4. Discussion

In this prospective study, we evaluated the prevalence of self-reported SDB symptoms suggestive of sleep-disordered breathing, as well as excessive daytime sleepiness during pregnancy, along with their associations with demographic and anthropometric factors. Our findings contribute to addressing a gap in Portuguese literature, where research on SDB in pregnant women is limited.

SBD symptoms were highly prevalent in our population, with participants reporting an average of 3.1 out of 10 possible symptoms, and some reporting up to seven. The most commonly reported symptoms were fatigue (65.4%), memory or concentration impairment (52.2%), and non-restorative sleep (50.5%). These findings reinforce prior literature suggesting that the physiological and hormonal changes in pregnancy contribute to sleep disruption and an increased vulnerability to SBDs, with potential implications for maternal well-being and pregnancy outcomes [2,7,9,10]. In line with these observations, 22.8% of participants exhibited excessive daytime sleepiness as defined by the ESS, corroborating earlier studies linking pregnancy to diminished sleep quality and increased daytime fatigue [2,7].

Comparative analyses of symptoms and clinical variables revealed several statistically significant associations. Snoring was associated with both older maternal age and higher pre-pregnancy BMI, further supporting a link between symptoms of SBD and known anatomical and metabolic risk factors [6]. Notably, symptoms such as snoring and non-restorative sleep consistently emerged as markers of sleep disruption in this population.

Regarding gestational age, certain symptoms varied clearly across trimesters. Although not all symptoms increased significantly over time, we observed a higher cross-sectional frequency of snoring, nocturnal dyspnea, fatigue, and daytime sleepiness in the third trimester. While some findings did not achieve statistical significance, the overall frequency trend indicates an increased burden of sleep-related symptoms during the third trimester [3,4,13]. Correspondingly, the proportion of participants with an ESS score greater than 10 rose from 18% in the first trimester to 32% in the third. This reinforces previous studies indicating that clinically relevant sleepiness escalates with advancing gestation [3,6]. These frequency trends likely reflect cumulative physiological changes in pregnancy, including fluid redistribution, upper airway narrowing, and rising progesterone levels, coupled with worsening sleep fragmentation over time [4,6]. Such progression should alert clinicians to consider sleep disruption in prenatal risk assessment and may warrant referral to specialty care for selected patients.

In our analysis of maternal age and its relations, the cohort exhibited a mean maternal age skewed toward older women. This trend mirrors the demographic reality in many Western developed countries, where delayed motherhood is increasingly common. Such a shift is linked to higher risks for both maternal and fetal health, including a greater predisposition to SBDs and other pregnancy-related comorbidities, such as gestational hypertension and diabetes [7]. Regarding the relation with self-reported SBD symptoms, although not statistically significant, some symptoms, like snoring and non-restorative sleep, appeared to show potential trends towards older age. Additionally, the self-reported SBD symptom score was more prevalent in older groups, possibly highlighting an age-related vulnerability to sleep disruption during pregnancy [12].

In examining symptom frequency by pre-pregnancy BMI, we found that snoring was significantly associated with higher BMI categories. This finding reinforces the correlation between elevated pre-pregnancy BMI and an increased risk of developing SBDs, such as OSA [3,5,6,15]. These results are consistent with previous research and underscore the necessity of closely monitoring these patient subgroups and ensuring timely referral for specialty consultation.

While comorbidities are generally infrequent in the pregnant population [16,17], a similar pattern was observed in our studied cohort, making statistically significant correlations difficult to establish. However, an exception was noted among women with diabetes. This subgroup demonstrated significantly higher odds of reporting snoring, witnessed apneas, and nocturnal dyspnea. These findings suggest that diabetes may increase susceptibility to symptoms related to SDB. The associations emphasize the importance of incorporating sleep symptom screening in prenatal assessments, particularly for women with metabolic risk factors.

This study presents several limitations. First, self-reported questionnaires, despite being accompanied by standardized written instructions, may have introduced reporting bias as well as selection bias being included a subset of women attending our tertiary center. Second, neither the ESS nor the symptom score used is formally validated for pregnant populations, which restricts the generalizability of the findings. Nevertheless, these tools are simple, low-cost, and easily repeatable throughout pregnancy, offering potential value for clinical screening pending further validation. Additionally, validated screening tools such as STOP-BANG or the Berlin questionnaire were not used. Although widely applied in the general population, their performance in pregnant populations remains suboptimal [9,10,11].

Third, due to the low prevalence of comorbidities in the pregnant population, our cohort included small comorbidity subgroups, which may limit the generalization of our results. Fourth, although symptom frequency and scores were explored across gestational age, participants were assessed only once, at different stages of pregnancy. Therefore, differences across gestational age reflect comparisons between different individuals rather than longitudinal changes within the same participant. Fifth, several of the most frequently reported symptoms, including fatigue, insomnia, non-restorative sleep, and memory/concentration impairment, are common in normal pregnancy and lack specificity for sleep-disordered breathing [8,9,10]. As a result, the composite symptom score may overestimate the burden of SBD-related symptoms and should be interpreted as a measure of overall sleep-related symptom burden rather than a proxy for confirmed SBD. Lastly, as stated, we did not conduct an objective sleep assessment, such as polysomnography or home sleep apnea testing, which can limit clinical interpretation of the results, and, therefore, the SBD symptoms we analyzed should not be interpreted as evidence of confirmed sleep-disordered breathing. Nevertheless, this study sets the stage for future investigations in this area alongside the development of pregnancy-specific screening tools, as emphasized in the recent literature [8,9].

## 5. Conclusions

In this cohort of pregnant women, symptoms related to SBD were highly prevalent and showed significant associations with maternal age, pre-pregnancy BMI, gestational progression, and comorbidities, particularly diabetes. Both the number of reported symptoms and the burden of excessive daytime sleepiness increased throughout pregnancy, with a marked rise in the third trimester.

In summary, our findings indicate that SBD symptoms are common during pregnancy and warrant a heightened suspicion for underlying SBDs, especially in later gestational stages. The associations observed between key symptoms and factors such as higher BMI, maternal age, gestational age, and comorbidities like diabetes suggest an increased risk for clinically relevant SBDs. These factors should be considered potential risk factors during obstetric evaluations.

These findings underscore the clinical significance of incorporating sleep assessments into prenatal care and emphasize the urgent need for validated screening tools tailored specifically for pregnant populations. Considering the potential maternal and fetal consequences of undiagnosed SBDs, early identification is crucial, particularly for high-risk groups, to enable appropriate referrals and timely interventions. Further research is necessary to validate symptom-based screening scores and to devise effective sleep testing strategies for this underexplored and sensitive population.

## Figures and Tables

**Table 1:** Descriptive statistics for continuous measures.

	All Participants (n *=* 289)
**Maternal Age, years, mean (SD)**	34.4 (4.9)
≤30 years, n (%)	64 (22.1)
31–35 years, n (%)	106 (36.7)
36–40 years, n (%)	84 (29.1)
>40 years, n (%)	35 (12.1)
**Gestational age, weeks, mean (SD)**	21.5 (9.1)
Pre-pregnancy BMI *, kg/m^2^, mean (SD)	23.6 (4.1)
Underweight (<18.5), n (%)	13 (4.5)
Normal weight (18.5–24.9), n (%)	194 (67.6)
Overweight (25.0–29.9), n (%)	54 (18.8)
Obese (≥30), n (%)	26 (9.1)
**Parity, n (%)**	
Nulliparous	126 (43.6)
Uniparous	83 (28.7)
Multiparous	80 (27.7)
**Trimester of pregnancy, n (%)**	
1st	70 (24.2)
2nd	130 (45.0)
3rd	89 (30.8)
**Maternal Age ≥ 40, n (%)**	47 (16.3)
**Comorbidities, n (%)**	31 (10.7) 23 (7.9) 14 (4.8) 10 (3.5)
Respiratory disease	
Asthma	
Hypertension	
Diabetes	
**ESS score ^#^, median [min–max]**	7 [0–20]
**SBD symptom score, median [min–max]**	3 [0–7]

Data are listed as mean (standard deviation), median [range], or number n (%). * n = 287; ^#^ n = 285. BMI, Body Mass Index; ESS, Epworth Sleepiness Scale.

**Table 2:** Frequency of reported sleeping breathing disorder symptoms in the overall group.

Symptoms	n (%)
Fatigue	189 (65.4)
Memory/Concentration Impairment	151 (52.2)
Non-Restorative Sleep	146 (50.5)
Insomnia	106 (36.7)
Daytime Sleepiness	102 (35.3)
Snoring	74 (25.6)
Nocturnal Movements	66 (22.8)
Behavioral Changes	42(14.5)
Nocturnal Dyspnea	16 (5.5)
Witnessed Apneas	8 (2.8)

## Data Availability

The data is available on demand from the corresponding author upon reasonable request.

## Supplementary Materials

The following supporting information can be downloaded at: https://www.mdpi.com/article/10.3390/medicina62050835/s1. Table S1: Associations between SBD symptoms and ESS, BMI, and maternal age; Table S2: Frequency of SBD-related symptoms by maternal age group.; Table S3: Frequency of SBD-related symptoms by pre-pregnancy BMI group.

## Abbreviations

The following abbreviations are used in this manuscript:

BMI	Body Mass Index
CI	Confidence Interval
ESS	Epworth Sleepiness Scale
IQR	Interquartile Range
OSA	Obstructive Sleep Apnea
OR	Odds Ratio
SBD	Sleep-related Breathing Disorder
SD	Standard Deviation
