# Supplementary material for: Sleep-Related Breathing Disorders and Pregnancy: Where We Stand and Where to Go
Source: Medicina (Kaunas). 2026 Apr 28;62(5):835. doi: 10.3390/medicina62050835 (PMC13208985; doi:10.3390/medicina62050835)
Supplement: Supplementary file 1 [file medicina-62-00835-s001.zip › Supplementary Material.pdf]

Supplementary Material

Table S1. Associations between SBD symptoms and ESS, BMI, and maternal age.

	**Symptom?**	**ESS (median)**	**Mann withney**	**Maternal age (mean)**	**Unpaired t-test**	**Gestational age (mean)**	**Mann withney**	**BMI (mean)**	**Unpaired t-test**
Fatigue	No	6	**<0.0001**	33.86		21.41		23.85	
Fatigue	Yes	8		34.67		21.57		23.43	
Memory/Concentration Impairment	No	6	**<0.0001**	33.68	**0.0185**	20		23.76	
Memory/Concentration Impairment	Yes	8		35.04		24		23.40	
Non-Restorative Sleep	No	6	**0.0193**	33.71	**0.0199**	23		23.86	
Non-Restorative Sleep	Yes	8		35.05		23		23.30	
Insomnia	No	7		34.43		23		23.78	
Insomnia	Yes	7		34.32		23		23.21	
Daytime Sleepiness	No	6	**<0.0001**	34.39		22	0.075	23.34	
Daytime Sleepiness	Yes	9		34.39		24		24.00	
Snoring	No	7		33.83	**0.0009**	22		22.88	**<0.0001**
Snoring	Yes	8		36.01		25		25.65	
Nocturnal Movements	No	7		34.3		22		23.38	0.1458
Nocturnal Movements	Yes	7		34.7		25		24.24	
Behavioral Changes	No	7		34.33		22	0.051	23.62	
Behavioral Changes	Yes	8		34.76		27		23.33	
Nocturnal Dyspnea	No	7	**0.0483**	34.31		23		23.53	
Nocturnal Dyspnea	Yes	9		35.75		26		24.29	
Witnessed Apneas	No	7		34.44		23		23.51	0.1261
Witnessed Apneas	Yes	9		33.75		22		25.81	

Table S2. Frequency of SBD-related symptoms by maternal age group.

Symptom	Snoring		Witnessed Apneas		Nocturnal Dyspnea		Daytime Sleepiness		Fatigue	
	Yes	No	Yes	No	Yes	No	Yes	No	Yes	No
**Age group (years)**										
**≤30**	11 (17.2%)	53 (82.8%)	1 (1.6%)	63 (98.4%)	2 (3.1%)	62 (96.9%)	21 (32.8%)	43 (67.2%)	41 (64.1%)	23 (35.9%)
**31-35**	24 (22.6%)	82 (77.4%)	4 (3.8%)	102 (96.2%)	4 (3.8%)	102 (96.2%)	39 (36.8%)	67 (63.2%)	65 (61.3%)	41 (38.7%)
**36-40**	25 (29.8%)	59 (70.2%)	3 (3.6%)	81 (96.4%)	8 (9.5%)	76 (90.5%)	32 (38.1%)	52 (61.9%)	62 (73.8%)	22 (26.2%)
**>40**	14 (40.0%)	21 (60.0%)	0 (0.0%)	35 (100.0%)	2 (5.7%)	33 (94.3%)	10 (28.6%)	25 (71.4%)	21 (60.0%)	14 (40.0%)
**P-value**	0,059		0.783*		0.299*		0,739		0,272	
**Symptom**	**Non-Restorative Sleep**		**Memory/Concentration Impairment**		**Nocturnal Movements**		**Insomnia**		**Behavioural Changes**	
	**Yes**	**No**	**Yes**	**No**	**Yes**	**No**	**Yes**	**No**	**Yes**	**No**
**Age group (years)**										
**≤30**	30 (46.9%)	34 (53.1%)	30 (46.9%)	34 (53.1%)	16 (25.0%)	48 (75.0%)	29 (45.3%)	35 (54.7%)	10 (15.6%)	54 (84.4%)
**31-35**	45 (42.5%)	61 (57.5%)	50 (47.2%)	56 (52.8%)	19 (17.9%)	87 (82.1%)	33 (31.1%)	73 (68.9%)	13 (12.3%)	93 (87.7%)
**36-40**	51 (60.7%)	33 (39.3%)	51 (60.7%)	33 (39.3%)	22 (26.2%)	62 (73.8%)	31 (36.9%)	53 (63.1%)	15 (17.9%)	69 (82.1%)
**>40**	20 (57.1%)	15 (42.9%)	20 (57.1%)	15 (42.9%)	9 (25.7%)	26 (74.3%)	13 (37.1%)	22 (62.9%)	4 (11.4%)	31 (88.6%)
**P-value**	0,066		0,205		0,508		0,326		0,678	

Values are presented as n (%). SBD, Sleep Breathing Disorder.
P-value indicates Chi-square or Fisher's exact test (*).

Table S3. Frequency of SBD-related symptoms by pre-pregnancy BMI group.

Symptom	Snoring		Witnessed Apneas		Nocturnal Dyspnea		Daytime Sleepiness		Fatigue	
	**Yes**	**No**	**Yes**	**No**	**Yes**	**No**	**Yes**	**No**	**Yes**	**No**
**BMI group**										
**Underweight**	1 (7.7%)	12 (92.3%)	0 (0%)	13 (100%)	1 (7.7%)	12 (92.3%)	4 (30.8%)	9 (69.2%)	8 (61.5%)	5 (38.5%)
**Normal**	38 (19.6%)	156 (80.4%)	4 (2.1%)	190 (97.9%)	9 (4.6%)	185 (95.4%)	63 (32.5%)	131 (67.5%)	125 (64.4%)	69 (35.6%)
**Overweight**	19 (35.2%)	35 (64.8%)	2 (3.7%)	52 (96.3%)	4 (7.4%)	50 (92.6%)	22 (40.7%)	32 (59.3%)	41 (75.9%)	13 (24.1%)
**Obese**	14 (53.8%)	12 (46.2%)	2 (7.7%)	24 (92.3%)	2 (7.7%)	24 (92.3%)	12 (46.2%)	14 (53.8%)	13 (50%)	13 (50%)
**P-value**	**0,0002**		0.275*		0.577*		0,417		0,138	

Symptom	Non-Restorative Sleep		Memory/Concentration Impairment		Nocturnal Movements		Insomnia		Behavioural Changes	
	**Yes**	**No**	**Yes**	**No**	**Yes**	**No**	**Yes**	**No**	**Yes**	**No**
**BMI group**										
**Underweight**	7 (53.8%)	6 (46.2)	9 (69.2%)	4 (30.8%)	0 (0%)	13 (100%)	5 (38.5%)	8 (61.5%)	2 (15.4%)	11 (84.6%)
**Normal**	98 (50.5%)	96 (49.5%)	97 (50.0%)	97 (50.0%)	40 (20.6%)	154 (79.4%)	71 (36.6%)	123 (63.4%)	28 (14.4%)	166 (85.6%)
**Overweight**	26 (48.1%)	28 (51.9%)	30 (55.6%)	24 (44.4%)	16 (29.6%)	38 (70.4%)	20 (37%)	34 (63.0%)	9 (16.7%)	45 (83.3%)
**Obese**	13 (50%)	13 (50%)	13 (50%)	13 (50%)	8 (30.8%)	18 (69.2%)	8 (30.8%)	18 (69.2%)	3 (11.5%)	23 (88.5%)
**P-value**	0,168		0,538		0,079		0,942		0.924*	

Values are presented as n (%). SBD, Sleep Breathing Disorder.
P-value indicates Chi-square or Fisher's exact test (*).
